# Silver Nanoparticles Biosynthesized With *Salvia officinalis* Leaf Exert Protective Effect on Hepatic Tissue Injury Induced by *Plasmodium chabaudi*

**DOI:** 10.3389/fvets.2020.620665

**Published:** 2021-02-04

**Authors:** Dina M. Metwally, Reem A. Alajmi, Manal F. El-Khadragy, Saleh Al-Quraishy

**Affiliations:** ^1^Department of Zoology, College of Science, King Saud University, Riyadh, Saudi Arabia; ^2^Department of Parasitology, Faculty of Veterinary Medicine, Zagazig University, Zagazig, Egypt; ^3^Department of Biology, Faculty of Science, Princess Nourah Bint Abdelrahman University, Riyadh, Saudi Arabia; ^4^Department of Zoology and Entomology, Faculty of Science, Helwan University, Cairo, Egypt

**Keywords:** sage leaf extract, antioxidant, gene expression, malaria, liver injury

## Abstract

Malaria is an important health problem in subtropical and tropical areas around the world. Infection with protozoan parasites of the Plasmodium genus, which grow inside host erythrocytes, causes malaria and may lead to morbidity and mortality. Liver tissue plays an important role in the pathogenesis of malaria and is closely involved in parasitic pre-erythrocytic development. Numerous published studies have demonstrated that the liver is not only the source of Plasmodium parasites prior to erythrocytic growth but is also a primary immune effector toward the blood stage of the malaria life cycle. Despite efforts to improve antimalarial drugs and vaccines, *Plasmodium species* that cause severe malaria are being detected increasingly frequently in endemic regions. In this study, *Salvia officinalis* (*S*. *officinalis*) leaf extract was employed to synthesize silver nanoparticles (Ag-NPs). This method is eco-friendly and represents a single-step technique for the biosynthetic process; therefore, it has attracted considerable attention. Accordingly, we biosynthesized Ag-NPs with extract of the *S. officinalis* leaf and examined the antimalarial activity of these nanoparticles in a murine model of *Plasmodium chabaudi* malaria (*P. chabaudi* malaria). Forty mice were chosen and classified into four types: infected group, healthy control, pretreated mice infected after treatment with 50 mg/kg of *S. officinalis* leaf extract-biosynthesized Ag-NPs for two weeks, and post-treated mice infected before treatment with 50 mg/kg of *S. officinalis* leaf extract-biosynthesized Ag-NPs (administered daily for 7 d). In this study, both pre-treatment and post-treatment with Ag-NPs produced a substantial reduction in parasitemia relative to the infected group. We investigated the antiplasmodial and hepatoprotective effects of *S. officinalis* leaf extract-biosynthesized Ag-NPs on *P. chabaudi*-induced inflammation and hepatic oxidative stress markers.

## Introduction

Malaria is triggered by parasitic disease caused by infection with protozoan parasites of the genus Plasmodium; hosts become infected through bites from infected female Anopheles mosquitoes (1). Malaria infection causes major health complications, which may lead to mortality and morbidity of the host. During host infection, sporozoites emitted from the mosquito's salivary glands penetrate the blood, and infected sporozoites enter the liver and destroy cells within seconds. The parasites undergo asexual reproduction (schizogony) and initiate division in the hepatocytes to make a massive pre-erythrocytic schizont containing several 100 merozoites. When infected hepatocytes rupture, merozoites enter the bloodstream and can attack erythrocytes (2).

The liver is a key effector site and can eliminate parasite-derived hemozoin during the blood stage of malaria through phagocytosis. One of the most significant pathological effects of malarial infections is oxidative damage (3). A valuable murine model for examining the role of the liver during malaria disease is *Plasmodium chabaudi* (*P. chabaudi*) since it has numerous shared characters to human pathogenic *P. falciparum* (4). The good achievement of chloroquine and its broad use throughout the many years ultimately prompted resistance to chloroquine in *P. falciparum* and *P. vivax*, the two parasite species liable for most instances of human malaria (5). Since early times, medicinal plants have been used to treat malaria as, the Chinese traditional treatment of malaria includes the use of *Artemisia annua* (Compositae) and its active compound, Artemisinin, which has a greater chemotherapeutic index than chloroquine and is effective in chloroquine-resistant strains of human malaria. These plants are promising anti-malarial agents (6).

Few research efforts have attempted to develop drugs from natural products as a means of identifying cost-effective and non-toxic drugs against malaria. Natural products are considered to be rich sources of chemical entities that may be employed to develop new operative drugs for the treatment of neglected diseases. Some metabolites from flavonoids, quinines, terpenes, and alkaloids can be easily utilized to treat diseases caused by protozoan parasites due to the scientific evaluation of medicinal plants (7).

Primaquine (PQ) and chloroquine (CQ) are the two widely used antimalarials in the tropical regions of the world (8). These drugs are used not only for the treatment of malaria but also for the prevention and prophylaxis, as well as for treatment of other diseases Some studies have indicated that antimalarials such as CQ and PQ inhibit cytochrome P450 mediated mixed function oxidase activities both *in vivo* and *in vitro* and lead to oxidative stress by decreasing non-enzymatic and enzymatic antioxidant defenses (9–12).

The antimalarial drugs that are commonly administered leave evidence of degradation after treatment, as demonstrated by memory decline in cerebral malaria (CM). Neurological complication, with the use of the CM model in mice, it has been shown that curcumin have potent activity as an immunomodulator and anti-oxidant activities which may alleviate CM and delay death of animals by about 10 days (13, 14).

Therefore, a number of researchers have specifically focused on the use of antioxidants, either alone or synergistically with antimalarial drugs, as a feasible treatment approaches to relieve Plasmodium-induced oxidative stress and alleviate its related health issues (15, 16).

However, practical implementation of this approach may provide contradictory results, as some antimalarials function by inducing oxidative stress. Vitamins C and E, deferoxamine, N-acetylcysteine, and folate are the most heavily researched antioxidants. Although some studies have evaluated the effectiveness on Plasmodium parasites of direct administration of antioxidants, other studies have employed adjunctive therapy with standardized antimalarials (17). Green synthesis nanoparticles are a great interest, since their large-scale application in the biomedical sector (nanomedicine). This is because particles synthesized by green technologies in the size range from 1 to 100 nanometers exhibit antioxidant, anti-inflammatory, and immunomodulatory activities. A distinctive feature of the nanoparticle's synthesis use plants (phytosynthesis) due to a higher rate of nanoparticle formation and contains a wide range of biomolecules such as, (poly) phenolic and flavonoids compounds (18, 19). Each antioxidant's therapeutic application in the management of malaria depends on the targeted element of malaria pathogenesis. These studies may establish a foundation for future research investigating the medicinal role of antioxidants in malarial pathogenesis (20). In this report, we describe the effectiveness of silver nanoparticles biosynthesized with leaf extract (*Salvia officinalis*) against an *in vivo* model of *Plasmodium chabaudi* malaria.

Scientific evaluation of medicinal plants has made it possible to use some metabolites from polyphenol and flavonoids for the treatment of diseases caused by protozoan parasites. Synthesis of silver nanoparticles (Ag-NPs) utilizing plant extracts has emerged as an alternative approach. There are a number of reasons for interest in green biosynthetic methods for Ag-NPs. They are cost-effective, simple, provide large quantities, are harmless and are environmentally friendly (7, 21). These methods are simple, have large yields and are cost-effective. Silver ions are reduced and stabilized by integrating organic compounds, including amino acids, proteins, tannins, saponins, phenolics, polysaccharides and nutrients, from plant/leaf extracts that have been characterized as having therapeutic benefits (22).

*Salvia officinalis* (sage) (family Lamiaceae) leaves are commonly employed as food flavorings and are a reliable resource for the rapid synthesis of Ag-NPs. Additionally, sage plants include tannins, volatile oils, triterpenes, diterpenes, flavones, steroids, and flavonoids. Sage leaf extracts have exhibited a variety of medicinal effects, including antioxidant effects, inhibitory effects, anti-hyperglycemic effects due to lipid peroxidation and anti-inflammatory properties (23–25).

Therefore, this study assessed the beneficial effect of employing Ag-NPs biosynthesized using *Salvia officinalis* leaf extract to treat a Balb/c mouse model of *P. chabaudi* malarial infection.

## Materials and Methods

### Plant Material and Extract Preparation

*Salvia officinalis* leaves were obtained from a sector in Riyadh, Saudi Arabia. The extract of the plant leaves was produced by combining 50 g of the leaves with 500 ml of sterile distilled water followed by boiling for 10 min. Next, the liquid was collected and filtered (Whatman No. 1 filter paper), and the filtrate was employed immediately to prepare Ag-NPs.

### Total Phenolic Content

The complete content of phenolic compounds in methanol extracts from *S. officinalis* was measured using the Folin-Ciocalteu technique as previously explained in (26). In brief, 0.2 mL of the test sample was placed in a test tube with a volume of 2.0 ml of sterile distilled water, preceded by 0.2 mL of unfiltered Folin-Ciocalteu reagent (Sigma-Aldrich, St. Louis, MO, US). The solution was mixed thoroughly and then allowed to stand for 6 min before the addition of 50 mL of 20% sodium carbonate. At room temperature (20°C), the color was allowed to form for 30 min, and then the absorbance was recorded at 760 nm by a spectrophotometer (PD 303 UV spectrophotometer, Apel Co., Limited, Saitama, Japan). A blank solution was formulated using 0.1 mL methanol instead of the extract. The measurement was analyzed with a gallic acid solution calibration graph and was presented as mg gallic acid comparable (eq.) per g of dry weight extract.

### Total Flavonoids

The colorimetric aluminum chloride technique was employed to measure the total content of flavonoids. Extricate of *S. officinalis* was obtained as in (27). In a test tube, 50 μL of the extract mixed with 4 mL of sterile distilled water was added to the mixture, and next, 0.2 mL of 5% NaNO_2_ solution and 0.2 mL of 10% AlCl_3_.6H_2_O was added. The mixture was allowed to stand for 6 min, and afterwards, 2 mL of 1 mol/L NaOH solution was added, and sterile distilled water was added to bring the total concentration of the mixture to 10 ml. The mixture was allowed to stand for the next 15 min, and absorbance was evaluated at 510 nm. A calibration curve was employed to assess the flavonoid content, and the measurements were presented as mg eq. rutin on dry weight per g.

### Synthesis of Silver Nanoparticles (Ag-NPs)

Green Ag-NPs were synthesized through the biosorption of Ag+ using the clean suspension of *Salvia officinalis* extract. Five milliliters of the *S. officinalis* sample was incorporated drop-by-drop into the AgNO_3_ alkaline solution (50 mL, 0.1 mM/mL) and mixed for 30 min at 45–55°C. Next, the mixture was ultrasonicated for 3 h. The color of the silver nitrate changed from colorless solution to brownish color, implying the development of Ag-NP. Dialysis of the retained AgNO_3_ was performed at 4°C toward deionized water. Using a zeta sizer (ZEN 3600, Malvern, UK), the developed Ag-NPs were characterized employing transmission electron microscopy (TEM) (JEM-1011, JEOL, Akishima, Japan). Additionally, a UV-Vis spectrophotometer was employed to verify green Ag-NP formation within 200–1,000-nm wavelengths. Using 1 cm of aligned quartz cells, the absorbance spectrum was measured using a PerkinElmer Lambda 40 B double-beam spectrophotometer. The consistency of the Ag-NPs was evaluated by monitoring the solution color at 4°C in the refrigerator after 20, 40, 50, and 60 d of processing.

### Infection of Mice

In this research study, female BALB/c mice (*n* = 40; 8 weeks of age) employed for the *in vivo* tests were collected from the animal house of the Female Center for Scientific and Medical Colleges in Riyadh, Saudi Arabia. The mice were reared in pathogen-free circumstances and provided a defined diet and water *ad libitum*. Cryopreserved *P. chabaudi* protozoa were passed through donor mice five times and were injected intraperitoneally into infected mice as initially described (28). The infused dose (10^5^ parasitized erythrocytes) was determined with the Neubauer chamber. The mice were intraperitoneally injected with 100 μl of phosphate sample buffer containing *P. chabaudi*-parasitized erythrocytes (28). Parasitemia was assessed in smudged-blood smears subjected to Giemsa staining, and complete erythrocytes were measured with a Neubauer chamber. This measurement used the calculation of the proportion of parasitemia: parasitemia (percent) = (number of infected erythrocytes/total number of erythrocytes) multiplied by 100.

### Experimental Design

The mice were randomly subdivided into four groups, with each group having 10 mice as follows:

**Group I:** Negative experimental group – mice were gavaged every day with 100 μL saline (7 days daily).
**Group II:** Positive control group – mice were infected by intraperitoneal injection of 10^5^ *P. chabaudi*-infected erythrocytes (29).
**Group III:** Pretreated group – mice were orally given silver nanoparticles biosynthesized using *S. officinalis* leaf extract at a dosage of 50 mg/kg 2 weeks before infection with 1 × 10^5^ *P. chabaudi*–infected erythrocytes (30, 31).
**Group IV:** Post-treated group, after 60 min infection, mice were treated with the silver nanoparticles biosynthesized using *S. officinalis* leaf extract oral form and continued to receive the nanoparticles at a dose of 50 mg/kg daily for 7 days (30, 32). On day 7 post-infection, all mice were sacrificed by CO_2_ oxygen starvation and dissected, and liver tissue samples were frozen at −80°C until they were processed for molecular and biochemical analysis.

### Enzymatic Antioxidant Status

Prepared liver homogenates were analyzed to characterize catalase (CAT) (33), glutathione reductase (GRd) (34) and glutathione peroxidase (GPx) (35).

### Oxidative Stress

To measure lipid peroxidation (LPO), homogenates of the liver were formulated in 50 mM Tris-HCl and 300 mM sucrose to measure the amount of malondialdehyde (MDA) produced using the thiobarbituric acid (TBA) methodology (36), but unlike the method described by (37, 38), the liver homogenates were employed to measure the concentrations of glutathione and nitrite/nitrate (nitric oxide; NO).

### Measurement of the Inflammation Markers Interleukin (IL)-1β and TNF-α

Quantitative observations of the concentrations of IL-1β (Cat. No. BMS606, Thermo Fisher Scientific) and TNF-α (Cat. No. EZMTNFA, Millipore) were made using enzyme-linked immunosorbent assay (ELISA) kits for mice according to the manufacturers' instructions.

### Mitochondrial Levels of Cytochrome C

An ELISA kit was employed to quantify the mitochondrial levels of cytochrome c (Abcam, ab110172) according to the manufacturer's instructions.

### Real-Time Polymerase Chain Reaction (RT-PCR)

Total RNA from liver tissue samples was obtained using the RNeasy Plus Minikit (Qiagen, Valencia, CA, USA). RevertAid H Minus Reverse Transcriptase (Fermentas, Thermo Fisher Scientific Inc., Waltham, MA, USA) was used to reverse-transcribe RNA. The PCR analyses were performed continuously utilizing an Applied Biosystems 7500 PCR System. The expression levels of the genes were calculated using Power SYBR Green (Life Technologies, Carlsbad, CA, USA) and the correlating threshold cycle method (39). Jena Bioscience GmbH (Jena, Germany) synthesized the PCR primers for the IL-1β and TNF-α genes. The specific primers were designed using the NCBI program Primer-Blast. Additionally, the mRNA levels were normalized to GAPDH in every sample. The primer sets are presented in Table 1.

**Table 1:** Primers used to amplify IL-1β and TNF-α genes.

**Name**	**Sense (5′-3′)**	**Antisense (5′-3′)**
IL-1β	GACTTCACCATGGAACCCGT	GGAGACTGCCCATTCTCGAC
TNF-α	AGAACTCAGCGAGGACACCAA	GCTTGGTGGTTTGCTACGAC
GAPDH	GCATCTTCTTGTGCAGTGCC	GATGGTGATGGGTTTCCCGT

### Statistical Analysis

The results are presented as mean values ± standard medium error (SEM). The results were calculated using single-way variance analysis (ANOVA). In keeping with the statistical packages for social sciences (SPSS version 20.0 IBM, Armonk, NY, USA), Duncan's test was employed as a *post-hoc* test to compare substantial differences between the groups.

## Results

Table 2 displays the total flavonoid and phenolic concentrations of the extract under investigation, and the levels were 12.234 ± 0.988 mg eq. gallic acid/g and 0.934 ± 0.053 mg eq. rutin/g.

**Table 2:** Experimental determination of total phenolic and flavonoid contents for *S. officinalis* extract.

**Parameters**	**Mean ± SEM**
Total phenols (mg eq. gallic acid/g sample)	12.234 ± 0.988
Total flavonoids (mg eq. rutin/g sample)	0.934 ± 0.053

**Figure 1:**
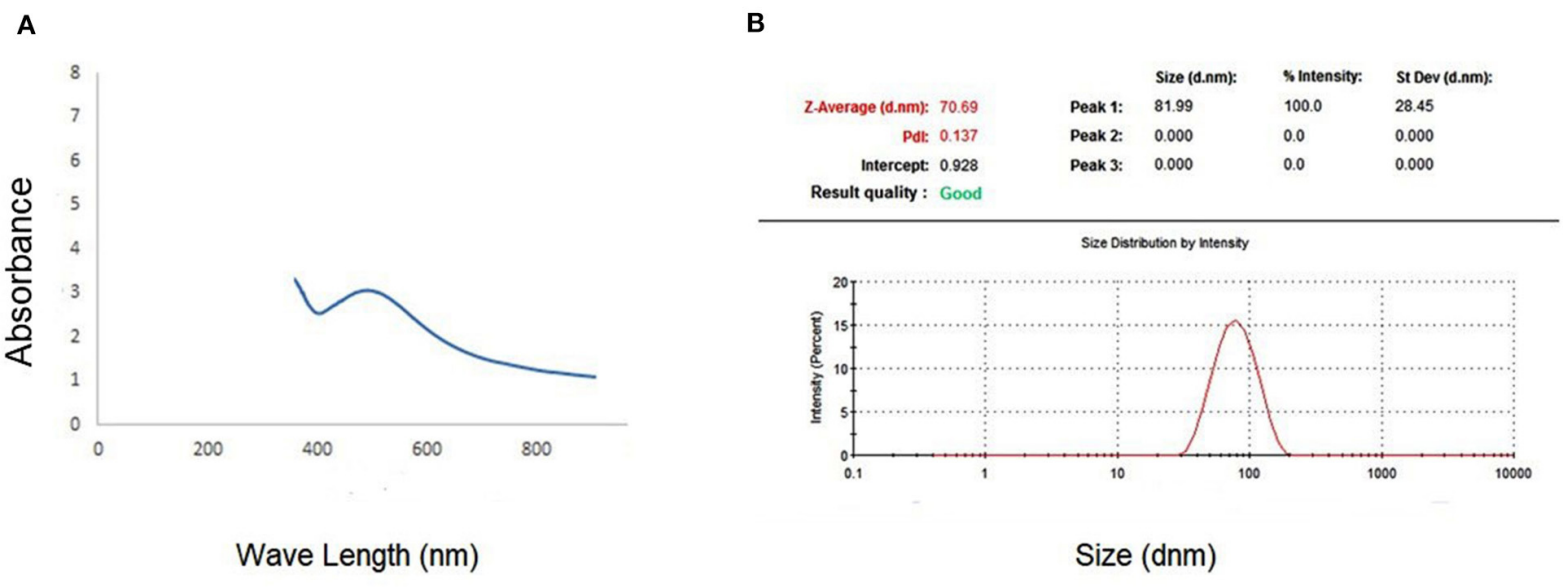
**(A)** Absorption coefficient of S-synthesized green silver nanoparticles. **(B)** Zeta sizer graph for calculating the mean size of green silver nanoparticles.

**Figure 2:**
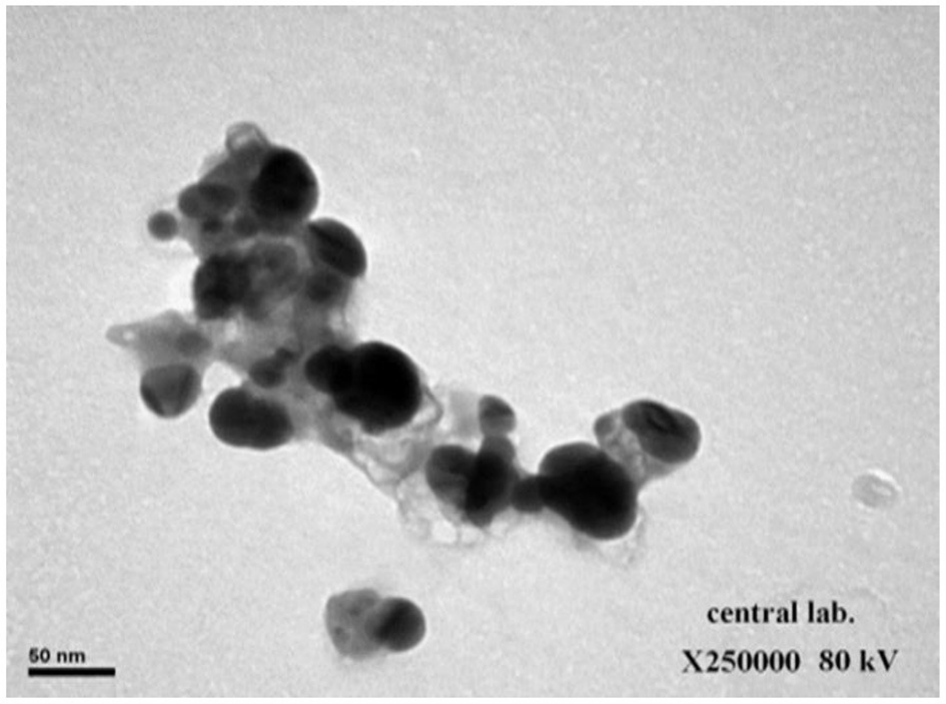
Transition electron microscopy (TEM) image showing synthesized green silver nanoparticles (scale bar: 50 nm).

The spectrophotometer measuring visible to UV light was employed to demonstrate the existence of Ag-NPs. The presence of a band at ~450 nm implies that Ag-NPs are formed (Figure 1A). Zeta analysis was performed to investigate the mean particle length within the nanometer (d.nm) to assess the number of synthesized silver nanoparticles. Figure 1B shows the dispersion of the size of the Ag-NPs. It was determined that the size of Ag-NPs varied between 51 and 226 d.nm with an optimal size of 81.99 d.nm. Additionally, the TEM image in Figure 2 shows that the majority of Ag-NPs were morphologically spherical.

Notably, the color of the Ag-NPs aqueous solution barely changed after 40 d of storage, indicating the stability of the Ag-NPs. After 60 d, the color varied to colorless.

Mice infected with *P. chabaudi* malaria showed some clinical signs and symptoms including stiff neck, increased muscle tone, ataxia, local paralysis, delirium, and blood urine. However, mice (pre-treatment and post-treatment with Ag-NPs biosynthesized with leaf extract of the *S. officinalis*) showed less or no clinical signs appeared.

**Figure 3:**
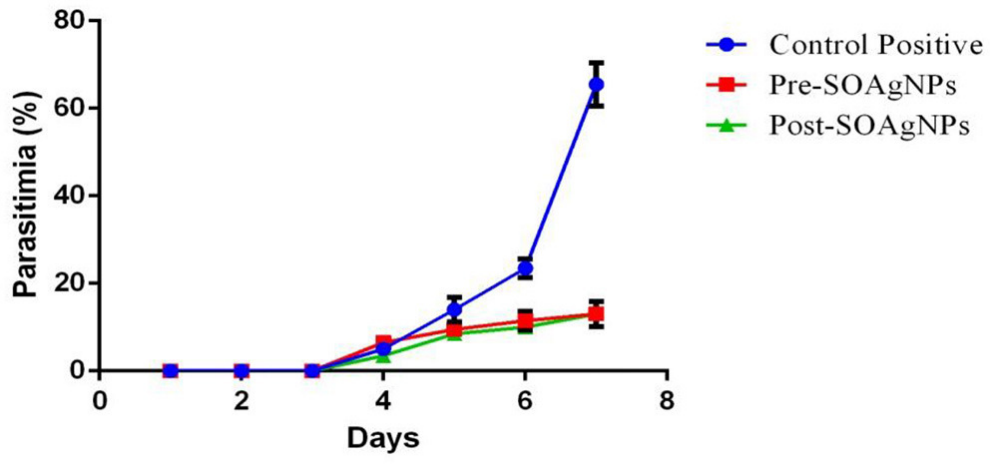
Treatment with *S. officinalis* leaf extract (Post-SOAg-NPs and Pre-SOAg-NPs) -biosynthesized AgNPs decreases the proportion of parasitemia at day 7 postinfection. Erythrocytes parasitized with *P. chabaudi*. Values are shown ± SEM.

Progressive increases in parasitemia were pronounced in infected mice after being inoculated with 10^5^ parasitized erythrocytes (Figure 3). Starting from day 4 postinfection with P. *chabaudi*, the parasitemia was decreased in the experimental groups treated with silver nanoparticles biosynthesized using *S. officinalis* leaf extract (pre-treatment and post-treatment), parasitemia were reduced ~5-fold on day 7 postinfection in comparison to the control group.

**Figure 4:**
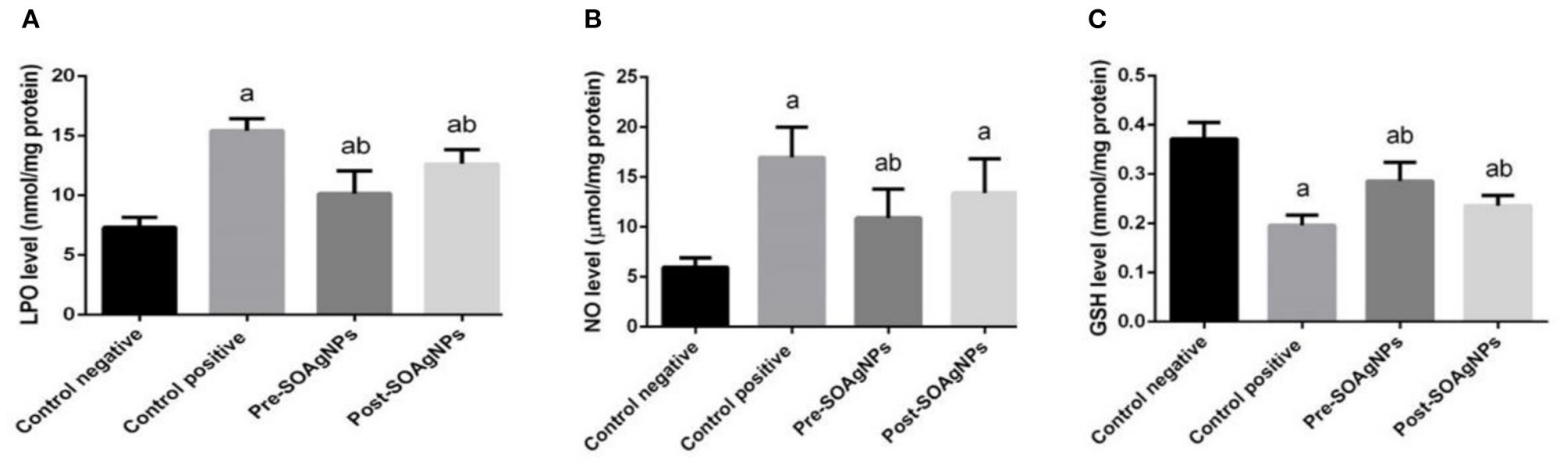
Effect of Ag-NPs biosynthesized by *S. officinalis* leaf extract on hepatic oxidative stress markers activities (Pre-SOAg-NPs and Post-SOAg-NPs): **(A)** malonaldehyde, **(B)** nitric oxide, and **(C)** glutathione. Values are mean ± SEM (*n* = 10); ^a^*p* < 0.05, significant change from –ve control group; ^b^*p* < 0.05, notable change from +ve control group.

In hepatic homogenates, the levels of lipid peroxidation (LPO) and nitrous oxide (NO) were assessed to estimate the impact of malarial infection on oxidative stress parameters (Figures 4A,B). *P. chabaudi* produced a significant (*p* < 0.05) increase in liver NO and LPO concentrations compared to the control group, whereas *S. officinalis*-biosynthesized AgNPs alleviated this increase significantly (*p* < 0.05).

*P. chabaudi* also stimulated hepatic oxidative stress, as depicted by a substantial decrease (*p* < 0.05) in the liver tissue quality of infected mice compared to the control group. This decrease in GSH levels was mitigated by post- and pre-infection treatment with AgNPs biosynthesized with *S*. *officinalis* leaf extract Figure 4C.

**Figure 5:**
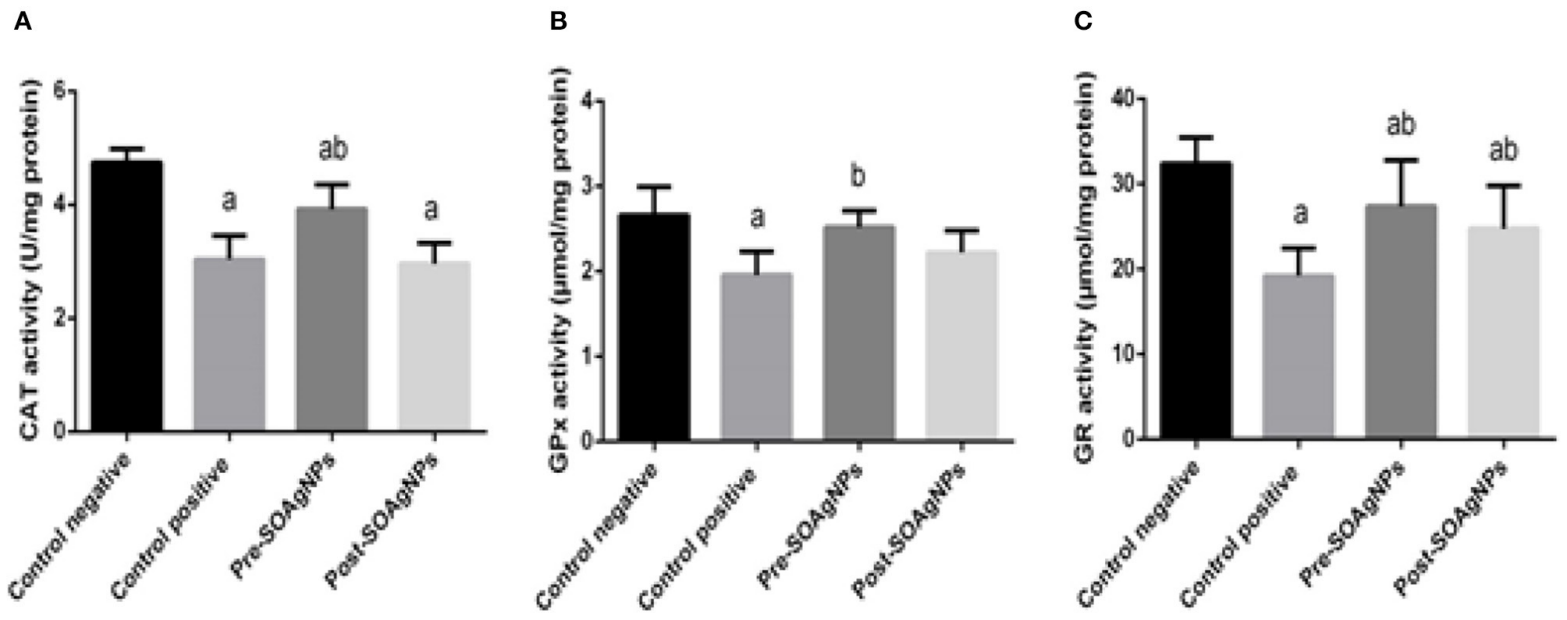
Effect of Ag-NPs biosynthesized by *S. officinalis* leaf extract on hepatic antioxidant enzyme activities (Pre-SOAg-NPs and Post-SOAg-NPs): **(A)** catalase, **(B)** glutathione peroxidase, and **(C)** glutathione reductase. Values are mean ± SEM (*n* = 10); ^a^*p* < 0.05, significant change from –ve control group; ^b^*p* < 0.05, notable change from +ve control group.

To analyze how *P. chabaudi* infection triggered oxidative stress in the tissue samples, possible changes in the antioxidant defense system were evaluated by determining the activities of the enzymes CAT, GPx, and GR. The repression of the activities of these enzymes by *P. chabaudi* infection was significantly alleviated by treatment with *S. officinalis* leaf extract-biosynthesized Ag-NPs (*p* < 0.05) (Figures 5A–C).

**Figure 6:**
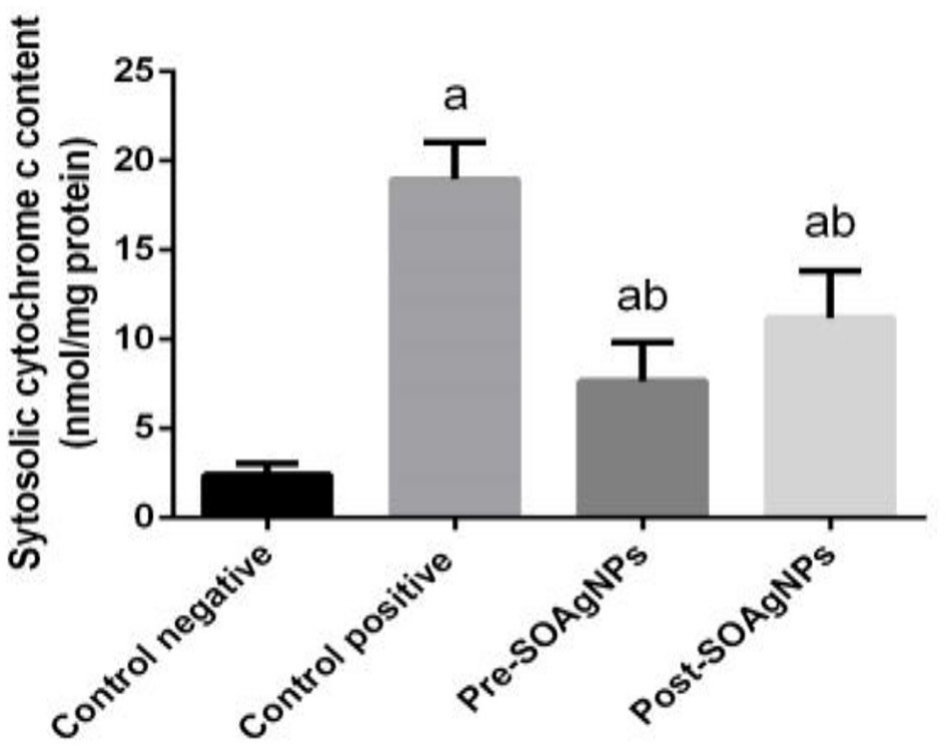
Effect of Ag-NPs biosynthesized by *S. officinalis* leaf extracts on hepatic cytochrome c activities (Pre-SOAg-NPs and Post-SOAg-NPs). Values are mean ± SEM (*n* = 10); ^a^*p* < 0.05, significant change from –ve control group; ^b^*p* < 0.05, notable change from +ve control group.

The level of cytochrome c activity in liver homogenates was increased in infected mice compared with the uninfected control (*p* < 0.05). Pre- and posttreatment with Ag-NPs biosynthesized with *Salvia officinalis* leaf extract significantly decreased cytochrome c activity compared with the untreated infected mice (Figure 6).

**Figure 7:**
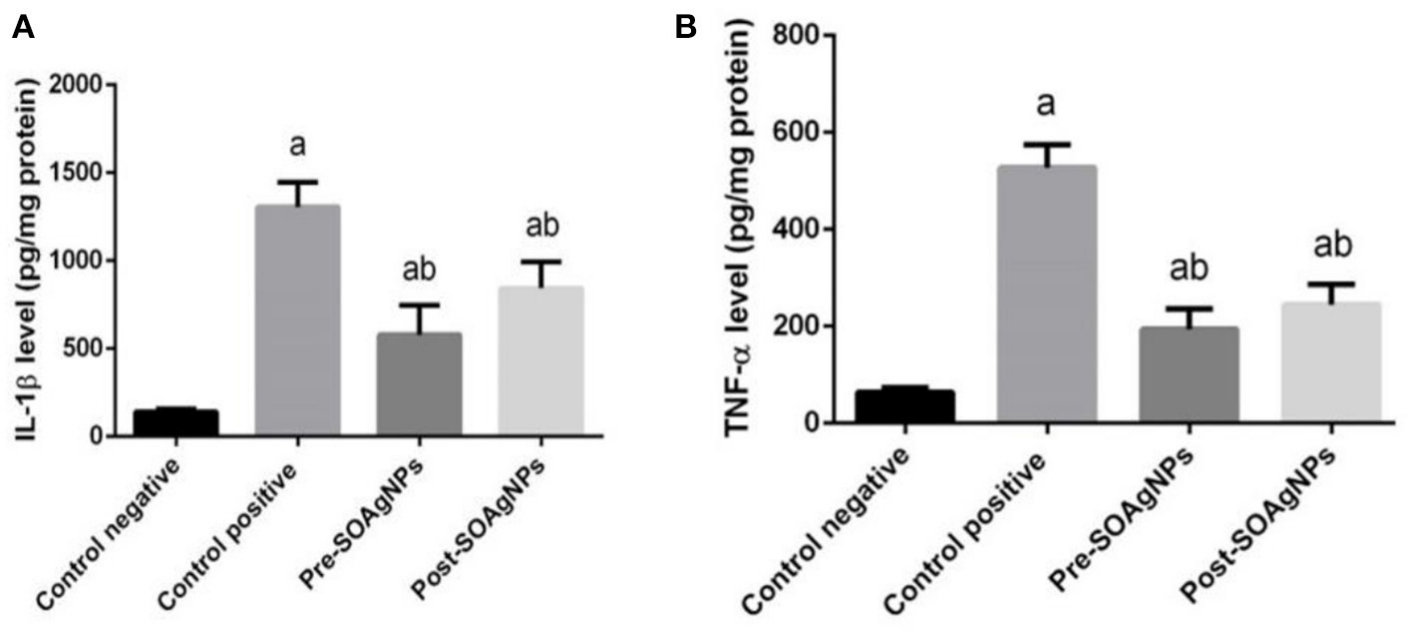
Effect of Ag-NPs biosynthesized by *S. officinalis* leaf extract on hepatic proinflammatory cytokines (Pre-SOAg-NPs and Post-SOAg-NPs). **(A)** IL-1β and **(B)** TNF-α. Values are mean ± SEM (*n* = 10); ^a^*p* < 0.05, significant change from –ve control group; ^b^*p* < 0.05, notable change from +ve control group.

Inflammation is characterized by the secretion of high levels of pro-inflammatory cytokines, such as IL-1β and TNF-α (Figures 7A,B) illustrated the effect of Ag-NPs biosynthesized by *S. officinalis* leaf extract on hepatic IL-1β and TNF-α that showed significantly increase in infected non-treated group level *p* < 0.05. But this increased that inhibited in Ag nanoparticles groups (Pre-SOAg-NPs and Post-SOAg-NPs).

**Figure 8:**
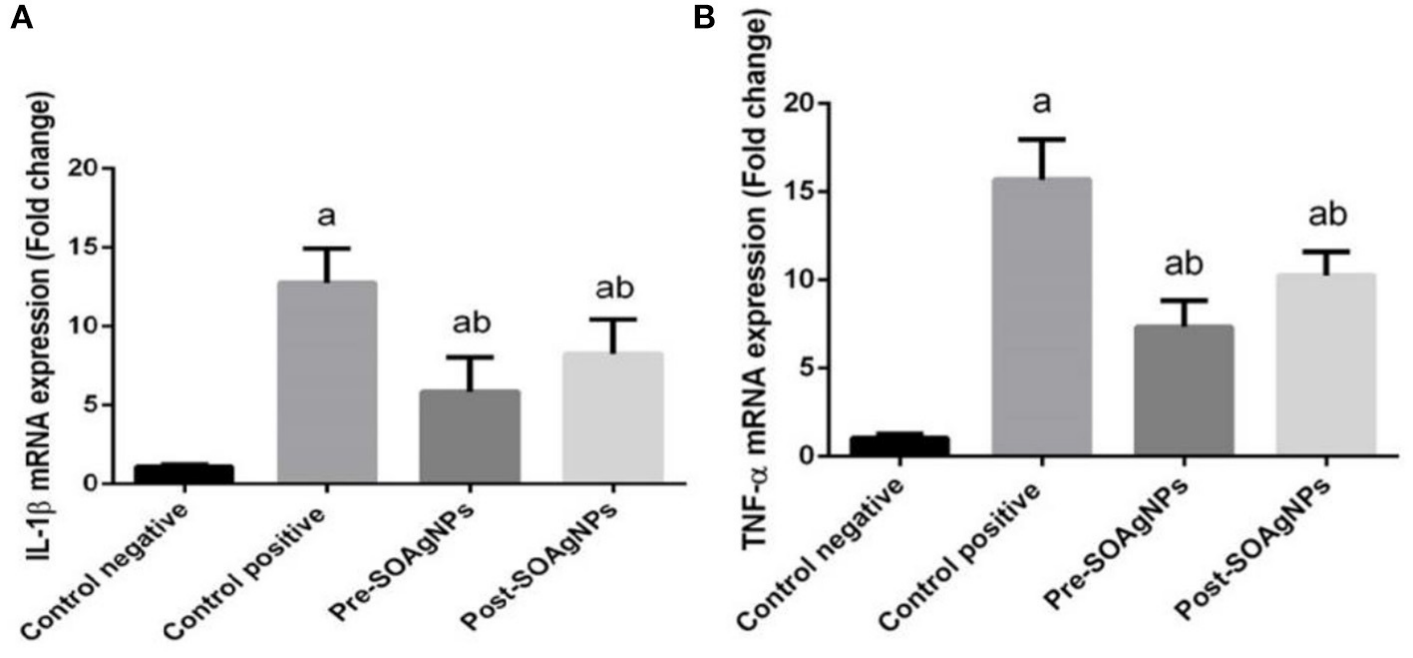
Effect of Ag-NPs biosynthesized by *S. officinalis* leaf extract (Pre-SOAg-NPs and Post-SOAg-NPs) on the hepatic proinflammatory cytokines **(A)** IL-1β and **(B)** TNF-α. Findings for the expression of genes are described as means ± SEM of triplicate assays, normalized to GAPDH and expressed as fold change (log2 scale), relative to mRNA levels in controls ^a^*p* < 0.05, significant change from –ve control group; ^b^*p* < 0.05, notable change from +ve control group.

To characterize the transcription of proinflammatory cytokine mRNA, this study determined that *P. chabaudi* triggered substantial expression of TNFα mRNA and IL-1β mRNA. In comparison, treatment with *S. officinalis* leaf extract-biosynthesized Ag nanoparticle groups (Post-SOAg-NPs and Pre-SOAg-NPs) triggered a marked downregulation of IL-1β and TNFα mRNA gene expression levels (Figures 8A,B).

## Discussion

Parasitic diseases remain a significant global health problem causing ~1 million deaths per year and affecting more than 1.7 billion people worldwide. Malaria may be the most life-threatening infectious disease of many diverse parasite infections (40). Due to the extreme complexity of parasite biochemistry and immunogenic variance, no efficient therapy has been recommended to date against malaria. As most commonly produced antimalarial drugs may be utilized to cure malaria, the underlying issue is drug resistance. Resistance to parasitic infections creates inconclusive or delayed parasitic infestation clearance from the patient's bloodstream after treatment with an antimalarial agent (41).

The liver is the very first pre-erythrocytic source of malarial infection and acts as receptor for malaria in the initial infection, where the liver epithelial system attacks the parasitized erythrocytes, potentially by phagocytosis. *P. chabaudi*-infected mice are characterized by parasitemia that reaches ~40% of infected erythrocytes and causes inflammation in the liver (42, 43).

One efficient strategy to combat parasite resistance is the detection of various sources of new antimalarial compounds, particularly from traditional plant sources. The use of plant species as production agents to be placed in silver nanoparticles has attracted attention because this method is notably rapid, has less environmental impact and represents a one-step method for a biosynthetic procedure (44).

Regarding the display of flavonoids and phenols, in the biosynthesis of these metal nanoparticles, *S. officinalis* plays an essential role. The synthesis of silver nanoparticles is reliable, replicable, and cost-effective, making it preferable to several other chemical methods (42).

*S. officinalis* leaf extract-biosynthesized Ag-NPs treatment (Pre-SOAg-NPs and Post-SOAg-NPs) may significantly decrease parasitemia, implying the removal of malaria parasites and their antimalarial influence. This clearance may be facilitated by both innate and acquired immune responses. Liver-formed cytokines are not only important for the local response in the liver but also may have an effect on the systemic response to blood-stage malarial infection (43, 45).

Nanobiotechnology's potential benefits began to emerge when nanoparticles were biosynthesized using bioactive ingredients, such as proteins, botanical extracts, vitamins, and biodegradable polymers (46, 47).

Nuclear factor-κB plays a pivotal role in immunity; this protein translocates to the nucleus and binds to the promoters of target genes as a transcription factor to πB motifs, and it activates the transcription of proinflammatory genes encoding IL-1β and TNF-α (48). The interaction of infected cells with or their encroachment of *P. chabaudi* products often causes IL-1β and TNF-α to induce CD8 ^+^ T and CD4 ^+^ T cell activation (46, 48, 49). Nuclear factor-κB has a seminal role in immunity, because it activates proinflammatory genes encoding iNOS, COX-2, TNF-α, IL-1β, and IL-6, it is activated by phosphorylation, ubiquitination and subsequent proteolytic degradation of the IκB protein by activated IκBkinase (IKK) (50). The liberated NF-κB translocate to the nucleus and binds as a transcription factor to κB motifs in the promoters of target genes, leading to their transcription. Aberrant NF-κB activity is linked with various inflammatory diseases, and most anti-inflammatory drugs suppress inflammatory cytokine expression by inhibiting the NF-κB pathway (51).

The evidence presented in this report shows a strong increase in proinflammatory cytokine rates triggered by *in vivo* infection with *P. chabaudi*. This process is initiated by omni-ubiquitination, phosphorylation, and consequent proteolytic deterioration of the I-B protein by I-B kinase (IKK) (52) activation. Nevertheless, *S. officinalis* leaf extract-biosynthesized Ag-NPs repressed the transcription of NF-κB in liver tissue; these results agree with (52), in which Ag-NPs were determined to maintain the repression of proinflammatory cytokines by reducing the phosphorylation of IκBα.

In this study, the leaves of *S. officinalis* are excellent sources for Ag-NPs biosynthesis; the development of Ag-NPs was indicated by a shift in color and stable solution. The color change is directly associated with the emergence of Ag-NPs, which can be attributed to the presence of reducing agents, such as flavonoids and phenols (53, 54). The TEM results demonstrate that the average particle size is ~81.8 d.nm and has a spherical form. Flavonoids and phenols are present in *S. officinalis* leaf extract, as well as other compounds. The official function of this extract is as the surface stabilizing component for Ag-NPs biosynthesis, as well as antioxidant and anti-inflammatory impacts. The biological components of Ag-NPs are encapsulated in *S. officinalis* extract. These characteristics agree with other *S. officinalis* leaf extract studies, which evaluated the extract for Ag-NPs synthesis using a 1 mM silver nitrate alkaline phase (55).

Liver diseases are among the most serious infections and represent a major public health issue worldwide; however, their management and treatment options are limited, despite major advances in medical technology. The pathophysiology of hepatic infections with the role of the inflammatory response and oxidative injury is well-known, and the chain reactions of the inflammatory response and oxidation reactions may therefore be slowed or blocked by promising therapeutic methods to avoid liver injury (53, 56).

The increased production of NO in reaction to the parasitic infection might be assumed to be an adverse outcomes of tissue damage and oxidative injury. The development of ROS in tissues and cells throughout numerous normal procedures indicates the pathophysiology of parasitic infections, such as *P. chabaudi* infection (37, 57). In the present study, *P. chabaudi* controlled Reactive oxygen species (ROS) synthesis in heavily infected mice in a manner that was inversely correlated with the extent of intracellular parasitization. NO radicals also were observed to play a significant role in the inflammatory process, which degrades biomolecules (58). Our findings show that levels of NO reliably increased in malaria-inoculated mice along with the severity of infection, and NO levels ultimately decreased after treatment with extract-biosynthesized Ag-NPs. Therefore, these results suggest a possible application in which nanoparticles may function as anti-inflammatory agents and thus safeguard the liver.

Recently, considerable experimental and clinical evidence has shown that the main apoptotic stimulus in various types of chronic and acute liver deterioration is chronic inflammation impacted by a discrepancy between the body's oxidant systems and antioxidants in favor of oxidants (59).

This phenomenon was suggested by increased lipid peroxidation correlated with lowered levels of the antioxidant enzymes, which included GPx, SOD, and GR. Antioxidant enzymes have been identified as being a mutually helpful system of defense against ROS. In this study, we reported that infection with *P. chabaudi* causes a substantial reduction in antioxidant enzyme activity. This decrease could result in higher superoxide radical absorption, which could facilitate induced lipid peroxidation. The reduction of enzymatic antioxidants after infection with *P. chabaudi* could be due to protein inhibition due to ROS, since oxidative damage can lead to the loss of a particular protein component, SOD, that catalyzes the H_2_O_2_ and O_2_ disproportionation of superoxide anion, since H_2_O_2_ is detrimental to cells (2, 60). Therefore, the organized activities of different cellular antioxidants in cell lines are crucial for the detoxification of free radicals. Mice infected with *P. chabaudi* reduced the activity of mouse liver antioxidant enzymes, which is consistent with the findings of other studies (61, 62). The results obtained with SOD, GPx, and GR demonstrate that the levels of ROS in the mouse liver were significantly changed upon infection, thereby affirming that free radicals and oxidative metabolism could play an essential role in the pathophysiology of liver injury (63).

GSH is the main antioxidant and the most abundant source of non-protein thiol found in liver cells, which acts as a substratum for some enzymes and plays a protective role in the metabolism of a large number of toxic agents. This substratum can act as a free radical trapping agent and safeguard cytochrome P450 by preventing lipid peroxidation (64). Nanoparticles increased substantially in this study and retained hepatic GSH activity after infection. Infection with *P. chabaudi* leads to a considerable decrease in the amount of glutathione, which may be an essential toxic effect alleviated by treatment with *S. officinalis* leaf extract-biosynthesized silver nanoparticles, which may be due to restoration of the GSH level. In this respect, researchers can use nanoparticles with antioxidant effects that are synthesized through the green synthesis method (where plant sublayers are used to prepare environmentally friendly nanoparticles that do not contain harmful chemical compounds). At present, using non-toxic precursors for synthesizing nanoparticles is largely regarded as a means of preventing biological hazards. The green synthesis method is believed to enhance the biocompatibility and performance of metal nanoparticles for biological applications.

Using the *S. officinalis* extract of leaves in silver nanoparticles was observed to attenuate malaria infection-induced apoptosis, and the amount of cytochrome c in the liver homogenates was quantified. ROS were shown to make the mitochondrial membrane more permeable and cause mitochondrial malfunction (47, 63).

The permeation of the mitochondrial membrane varies depending on the transition pore, resulting in cytochrome c being released from the mitochondria to the cytoplasm. Once set to release, cytochrome c may bind to apoptotic proteolytic enzyme-activating factor−1 (Apaf−1) in the cytosol, forming a complex that upregulates caspase 9 with enhanced expression of death-inducing variables (65, 66). Our results showed that in the infected mice, *S. officinalis* leaf extract-biosynthesized Ag-NPs treatment reversed the change in cytochrome c levels, and may potentially have prevented all detrimental occurrences, solubilized the oxygen-based reaction mixture produced in mitochondria, and stabilized the infection-induced mitochondrial membrane.

## Conclusions

Our results indicate that Ag-NPs biosynthesized from the *S. officinalis* leaf extract may be involved in the implementation of a new candidate therapeutic agent with greater efficacy in suppressing hepatotoxicity by modulating the cellular redox status while downregulating pro-inflammatory cytokine genes and inflammation. These results suggest that the potential mechanism of the anti-inflammatory properties of the *S. officinalis* leaf extract-biosynthesized silver nanoparticles was the intracellular blockade of inflammatory pathways and downregulation of proinflammatory cytokines.

## DRYAD Url

(https://datadryad.org/stash/share/S15z6f0OtTiUhHwjFOlzZLceHkDjK63EFpUHf0ntX4Y) Doi (https://doi.org/10.5061/dryad.1ns1rn8s5).

## Data Availability Statement

All datasets generated for this study are included in the article/supplementary material.

## Ethics Statement

The animal study was reviewed and approved by this research (IRB Number: KSU-SE-20-36) received approval from King Saud University's (Saudi Arabia) Organizational Committee for Postgraduate Research and Studies.

## Author Contributions

DM, RA, ME-K, and SA-Q: conceptualization, data curation, formal analysis, funding acquisition, investigation, methodology, project administration, resources, software, supervision, validation, visualization, writing original draft, writing review and editing. All the authors contributed equally to this work.

## Conflict of Interest

The authors declare that the research was conducted in the absence of any commercial or financial relationships that could be construed as a potential conflict of interest.
